# Improved gross primary production estimation in rice fields through integrated multi‐scale methodologies

**DOI:** 10.1002/pei3.10109

**Published:** 2023-06-10

**Authors:** Bora Lee, Hyojung Kwon, Peng Zhao, John Tenhunen

**Affiliations:** ^1^ Warm Temperate and Subtropical Forest Research Center National Institute of Forest Science Seogwipo‐si 63582 South Korea; ^2^ Forest Ecosystems & Society Oregon State University Covallis Oregon USA; ^3^ Department of Health and Environmental Sciences, Plant Ecology Jiaotong‐Liverpool University Xi'an, Suzhou P.R. China; ^4^ Plant Ecology University of Bayreuth Bayreuth Germany

**Keywords:** carbon uptake, gross primary production, photosynthesis model, rice paddy

## Abstract

Understanding productivity in agricultural ecosystems is important, as it plays a significant role in modifying regional carbon balances and capturing carbon in the form of agricultural yield. This study in particular combines information from flux determinations using the eddy covariance (EC) methodology, process‐based modeling of carbon gain, remotely (satellite) sensed vegetation indices (VIs), and field surveys to assess the gross primary production (GPP) of rice, which is a primary food crop worldwide. This study relates two major variables determining GPP. The first is leaf area index (LAI) and carboxylation capacity of the rice canopy (Vc_uptake_), and the second being MODIS remotely sensed vegetation indices (VIs). Success in applying such derived relationships has allowed GPP to be remotely determined over the seasonal course of rice development. The relationship to VIs of both LAI and Vc_uptake_ was analyzed first by using the regression approaches commonly applied in remote sensing studies. However, the resultant GPP estimations derived from these generic models were not consistently accurate and led to a large proportion of underestimations. The new, alternative approach developed to estimate LAI and Vc_uptake_ uses consistent development curves for rice (i.e., relies on consistent biological regulations of plant development). The modeled GPP based on this consistent development curve for both LAI and Vc_uptake_ agreed with *R* ^2^ from 0.76 to 0.92 (within the 95% confidence interval). The results of this study demonstrate that improved linkages between ground‐based survey data, eddy flux measurements, process‐based models, and remote sensing data can be constructed to estimate GPP in rice paddies. This study suggests further that the conceptual application of the consistent development curve, such as the combining of different scale measurements, has the potential to predict GPP better than the common practice of utilizing simple linear models, when seeking to estimate the critical parameters that influence carbon gain and agricultural yields.

## Graphical abstract

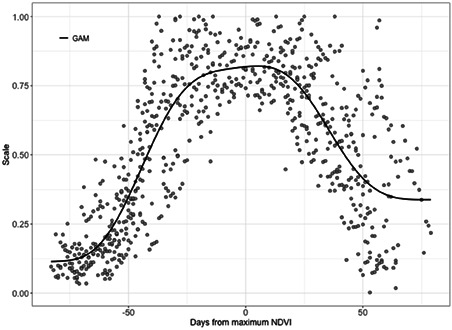

## 1 INTRODUCTION

Gross primary production (GPP) represents the total carbon assimilation by vegetation through photosynthesis, playing a vital role in crop yield, carbon balance, and ecosystem functioning (Beer et al., 2010; Moureaux et al., 2008; O'Sullivan et al., 2020). GPP refers to the total uptake of carbon dioxide in photosynthesis or CO_2_ assimilation, which drives subsequent ecosystem processes, including plant growth and agricultural yield (Schulze, 2006). Accurate estimation of GPP is essential for understanding the impacts of climate variability on agricultural productivity and ensuring global food security (Huntzinger et al., 2017; Liu et al., 2020). Rice (*Oryza sativa* L.) is a staple food crop for over 50% of the world population and occupies approximately 150 million hectares of land, making the accurate estimation of rice GPP critical (Lindner et al., 2016; Ray et al., 2013).

In recent years, significant advancements have been made in estimating GPP using eddy covariance (EC) methods, process‐based models, and satellite remote sensing (Baldocchi, 2014; Li et al., 2018; Peng & Gitesol, 2011, 2012; Zhang et al., 2018). The increased availability of high‐resolution satellite data, such as Landsat‐8 and Sentinel‐2, as well as rapid developments in UAV technology, has improved the spatial resolution of GPP estimations (Chaves et al., 2020; Wulder et al., 2019). However, estimating GPP in agroecosystems like rice paddies remains a challenge due to the incompatibility of spatial scales of flux measurements and satellite imagery resolution, as well as the influence of local climate, water availability, and varied management practices (Wattenbach et al., 2010; Yu et al., 2017).

Recent studies have reported the integration of advanced remote sensing techniques, such as near‐infrared vegetation indices (NIRv) and near‐infrared vegetation index with photosynthesis (NIRvP), to better capture rapid changes in crop development and improve GPP estimation (Badgley et al., 2017; Luo et al., 2019). The main goal of this study is to assess GPP at rice paddies in Asia and Europe, integrating recent advancements in remote sensing, process‐based modeling, and eddy covariance measurements to provide a more accurate estimation of rice GPP (Bouman et al., 2007; Gan et al., 2021). While recent research has made significant advancements in GPP monitoring and modeling, the current study differentiates itself by integrating multiple data sources and techniques for estimating GPP in rice paddies, addressing the unique challenges posed by this agroecosystem. By combining ground‐based survey data, eddy flux measurements, process‐based models, and remote sensing data, this study offers a more accurate and comprehensive approach for GPP estimation in rice paddies (Ogutu et al., 2013; Peng & Gitesol, 2012). This integrative approach not only enhances the understanding of the impacts of climate variability on agricultural productivity but also provides valuable insights for informed crop management decisions and ensuring global food security (Revill et al., 2013).

In this study, we use the physiologically based PIXGRO canopy model, combined with high‐resolution remote sensing data and eddy covariance measurements, to estimate rice paddy GPP (Li et al., 2018; Tenhunen et al., 2009). We aim to establish new relationships between remotely sensed vegetation indices, such as NDVI, and GPP and biomass, enabling improved parameterization of process‐based models at the landscape scale (Gamon et al., 1995; Nemani et al., 2003; Wang et al., 2004).

The objectives of this study are:

1. To use NDVI to examine rice paddy phenology and establish relationships between observed LAI and vegetation indices (Lee et al., 2016)
2. To estimate the critical PIXGRO parameter Vc_uptake_ of rice paddies from EC daily values in conjunction with leaf area index (LAI), which controls for canopy carbon fixation in response to multiple environmental factors (Owen et al., 2007)
3. To define the relationship between the daily NDVI and modeling parameter Vc_uptake_, establishing best‐fit models to estimate GPP in the absence of observations of CO_2_ exchange and LAI.

The process‐based model focused on the physiological process‐based canopy sub‐model of PIXGRO (Tenhunen et al., 2009) that links flux observations from the eddy covariance studies with ecosystem physiology represented as the capacity for CO_2_ exchange (i.e., canopy content and activity of Rubisco) and plant phenology (i.e., leaf area index). PIXGRO is designed as a tool for bridging between measured gas exchange fluxes, derived parameters for carboxylation capacity, seasonal changes in biomass and structure in the case of herbaceous and crop plants, and crop yields, considering specific ecophysiological behavior of individual species (Adiku et al., 2006). The canopy sub‐model of PIXGRO (hereafter referred to as “canopy model”) calculates the dynamics of whole‐ecosystem CO_2_ and H_2_O exchange (Reichstein, 2001).

We hypothesize that combining the physiologically based PIXGRO canopy model with GPP obtained from EC rice paddy sites allows for a more accurate definition of the seasonal course of the critical parameter Vc_uptake_. The seasonal course of the parameter Vc_uptake_, together with meteorological data, enables a more efficient reproduction of observed data that then helps more clearly define GPP. We also hypothesize that Vc_uptake_, estimated using seasonally observed LAI, performs better in estimating GPP than Vc_uptake_ using a constant LAI. Estimating GPP through this best‐fit model for Vc_uptake_ and LAI in dependence on NDVI subsequently provides more accurate GPP predictions (Li et al., 2008, 2018; Zhang et al., 2018).

## 2 METHODS AND MATERIALS

### 2.1 Study sites

The analysis was conducted using available rice paddy data from South Korea (Haean Catchment) and Japan (Mase) in Asia and Spain (El Saler‐Sueca) in Europe. Data used in the analysis cover the year of 2010 for Haean (HK), 2002–2005 for Mase (MSE), and 2007–2008 for El Saler‐Sueca (ESES2). All of the Asian sites were under the influence of a monsoon climate, with >50% of annual precipitation occurring during the summer monsoon period. Meteorological conditions and site characteristics are summarized in Table 1. The detailed information on landscape structure is described in Seo et al. (2014).

**TABLE 1:** Site information of rice paddy sites at Haean, South Korea, Mase, Japan, and El Saler‐Sueca, Spain.

		Site information			Meteorological condition				Planting	Harvest	Max LAI (DOY)
Site	Year	Country	Coordinates	Elevation (m)	Total R_g_ (MJ/m^2^/d)	Mean T_a_ (°C)	Mean VPD (kPa)	P (mm)			
Haean	2010	S. Korea	38.29 N, 128.13 E	446	1712.5 (6.79)	20.4 (4.09)	0.42 (0.27)	1165.2	144	290	5.82 (219)
Mase	2002	Japan	36.05 N, 140.03 E	13	2355.9 (7.70)	22.0 (4.41)	0.61 (0.32)	593.0	122	262	5.45 (204)
Mase	2003	Japan	36.05 N, 140.03 E	13	2049.2 (6.60)	20.3 (3.32)	0.51 (0.23)	545.4	122	262	5.06 (224)
Mase	2004	Japan	36.05 N, 140.03 E	13	2384.4 (7.28)	22.7 (4.29)	0.63 (0.31)	546.9	123	254	4.91 (209)
Mase	2005	Japan	36.05 N, 140.03 E	13	2236.6 (6.43)	21.8 (4.45)	0.54 (0.24)	646.6	122	256	4.38 (207)
El Saler	2007	Spain	39.28 N, −0.32 E	10	3223.7 (5.88)	22.8 (1.93)	0.55 (0.23)	437.4	134	270	5.67 (200)
El Saler	2008	Spain	39.28 N, −0.32 E	10	3262.5 (6.40)	22.1 (2.81)	0.51 (0.19)	121.2	132	278	6.07 (219)

*Note*: Total Rg is total global radiation (in parentheses, standard deviation), mean Ta is mean air temperature (in parentheses, standard deviation), total P is total rainfall, planting, and harvest date (day of year–DOY), maximum LAI, and DOY of maximum LAI. Meteorological condition considers the period of crop growth (from the planting to the harvest).

#### 2.1.1 Haean, South Korea (HK)

Haean Catchment is a typical erosion mountain basin in South Korea located northeast of Chuncheon, Gwangwon Province in Yanggu County (38° 17′ N, 128° 08′ E, 450–1200 m above sea level (a.s.l.)). The total area of the catchment is 64 km^2^, consisting of 58% forested mountain area, 30% agricultural area, and 12% as residential, riparian, field margins, and farm road area according to land surveys (Arnhold et al., 2014). The agricultural area is characterized as a mosaic patchwork of fields, with a dominance of dry‐land fields (22% of the total area) and rice paddy fields (8%) as the remaining area. Rice paddies (*Oryza sativa* L., cv. Odae) are cultivated at <500 m a.s.l. in the catchment (Choi et al., 2010).

Measurements of CO_2_ exchange with the eddy covariance (EC) method were conducted in 2010 at rice paddy site. Rice was transplanted on day of year (DOY) 144 and harvested on DOY 290, the EC system ran during three time periods at the rice paddy site (i.e., DOY 177–186, 203–223, and 242–274).

#### 2.1.2 Mase, Japan (MSE)

The Mase site is located in the rural area (36° 03′ 14′′ N, 140° 01′ 38″ E, 15 m a.s.l.) of Tsukuba City in Central Japan. The rice paddy (*Oryza sativa* L.) was ca. 2 km^2^ and was managed as a single rice‐cropping field following practices common in the area (Saito et al., 2005). In this study, the EC flux and meteorological data from 2002 to 2005 obtained from AsiaFlux (https://db.cger.nies.go.jp/asiafluxdb/) were included.

#### 2.1.3 El Saler‐Sueca, Spain (ESES2)

The El Saler Sueca site is located in the protected wetland area of La Albufera Natural Park in the Valencia region of Spain (39° 16′ 32′′ N, 0° 18′ 55″ E, 10 m a.s.l.). El Saler, which is in a sub‐arid Mediterranean climate, experiences hot summers with almost no rain and cold winters with substantial rainfall. The rice paddy was ca. 15 km^2^ and had been managed in same way for 200 years (Kutsch et al., 2010; Moors et al., 2010). The EC flux, meteorological data, and LAI of El Saler Sueca in 2007 and 2008 were obtained from CarboEurope cropland network (http://www.carboeurope.org/; Table 1).

### 2.2 Process‐based model

#### 2.2.1 Canopy model description

The canopy model for predicting GPP in comparison to data from EC sites is designed to calculate short‐term ecosystem CO_2_ exchange (Reichstein et al., 2003; Tenhunen et al., 1994; Wang et al., 2003). The model is single‐layered model. It estimates light interception and CO_2_ exchange rates of canopy foliage for sun and shaded light classes half‐hourly, which is then compared to EC measurements (Owen et al., 2007). The model is driven by meteorological data, for example, global radiation (R_g_), air temperature (T_a_), vapor pressure deficit (VPD), wind speed, air pressure, and atmospheric CO_2_ concentration, and requires estimated values for LAI. Total shortwave radiation on the sunlit leaves is the sum of direct, sky diffuse, and multiple scattered radiation, whereas on the shaded leaves, it is only the sum of sky diffuse and multiple scattered radiation (see Equations (2), (3), (4), (5) in Owen et al., 2007). The foliage orientation function (G) was set at 0.5 and the influence of clumping (Ω) at 0.9 for croplands in these equations. In order to account for the effect of the canopy on light interception, we expanded LAI to plant area index (PAI), which is the sum of LAI and stem area index (SAI) (i.e., PAI = LAI + SAI). SAI of the crop is calculated as 14% of LAI, whereas SAI of the rice is set at 0.01 (see details in Owen et al., 2007).

Simulation of gross photosynthesis follows Farquhar and Caemmerer (1982), as modified for practical field applications by Harley and Sharkey (1991). It is based on Ribulose‐1,5‐bisphosphate‐carboxylase‐oxygenase (Rubisco) enzyme reactions, where the rate of CO_2_ fixation is limited by either the regeneration of Ribulose‐1,5‐biphosphate (RuBP) at low light intensity and/or high internal CO_2_ concentration or by Rubisco activity and CO_2_/O_2_ concentration at saturated light and low internal CO_2_ concentration. The key parameter of the model is Rubisco maximum carboxylation rate (Vc_max_) at 25°C, while all temperature dependencies are fixed in relation to this rate. When comparing predicted GPP to EC measured values, a best fit is obtained for this key parameter. RuBP reduction capacity, dark respiration capacity, and light utilization efficiency of the canopy are assumed to be proportional to Vc_max_. Given that fixed temperature dependencies and process proportionalities are used and that assumptions are made about canopy structure and light interceptions, a lumped parameter, Vc_uptake_, that is assumed to control overall carbon fixation rather than direct enzyme related parameter Vc_max_ is obtained from the statistical fitting procedure.

The model formulation follows Farquhar and Caemmerer (1982) further; net photosynthesis (*P*_net_) is obtained using

(1) $$P_{\mathrm{net}}=\left(1-\frac{\Gamma^{*}}{c_{i}}\right)\min\left(w_{c}:w_{j}\right)-0.5R_{d}$$

where $\Gamma^{*}$ is CO_2_ compensation point in the absence of mitochondrial respiration, *w*_c_ is the carboxylation rate supported by Rubisco enzyme, *w*_j_ is the carboxylation rate supported by the actual electron transport rate, and *R*_d_ is the respiration occurring in mitochondria without light. *c*_i_ is the internal CO_2_ concentration based on Fick's Law for molecular diffusion of CO_2_ through the stomata and boundary layer and is calculated from the following equation.

(2) $$c_{i}=\mathrm{cs}-\frac{1.6P_{\mathrm{net}}}{g_{s}}$$

where *c*_s_ is the CO_2_ concentration at the surface of the leaf and *g*_s_ is the stomatal conductance according to modified Ball‐Berry equation (Ball et al., 1987; Harley & Sharkey, 1991).

(3) $$g_{s}=g_{s,\min}+\text{gfac}\frac{\left(P_{\mathrm{net}}R_{d}\right)\mathrm{rH}}{c_{s}}$$

where $g_{s,\min}$ is the minimum stomatal conductance, rH is relative humidity, and gfac is a constant representing stomatal sensitivity in relation to CO_2_ assimilation. It has been evaluated for different species from chamber experiments (Sala & Tenhunen, 1994, 1996; Tenhunen et al., 1990).

*w*_c_ is the carboxylation rate supported by Rubisco enzyme, calculated as:

(4) $$w_{c}=\frac{\mathrm{Vc}\;c_{i}}{c_{i}+K_{c}\left(1+O/K_{O}\right)}$$

where Vc is the maximum rate of carboxylation, *K*_c_ is the Michaelis constant for carboxylation, *K*_o_ is the Michaelis constant for oxygenation, and *O* is the oxygen concentration of the air [210 cm^3^ O_2_ (L air)≠1]. As the dependency of temperature, Vc is calculated as:
(5)

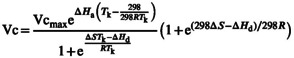

where Vc_max_ is the maximum rate of carboxylation capacity at 25°C, *H*_a_ is the activation enthalpy of carboxylation, *T*_k_ is the estimated the leaf temperature in the current model iteration step, *R* is the universal gas constant, *S* is an enthalpy term for deactivation, and *H*_a_ is the deactivation enthalpy of carboxylation. *w*_j_ is calculated as:

(6) $$w_{j}=\frac{P_{m}c_{i}}{c_{i}+2.0\Gamma^{*}}$$

where *P*_m_ is the maximum potential rate of RuBP production. *P*_m_ is calculated following the Smith equation (cf. Tenhunen et al., 1976):

(7) $$P_{m}=\frac{\text{alpha}I}{\sqrt{1+\left(\frac{{\text{alpha}}^{2}I^{2}}{P_{\mathrm{ml}}^{2}}\right)}}$$

where alpha is the average leaf light utilization efficiency without photorespiration, *I* is the incident PPFD, and *P*_ml_ is the CO_2_ and light saturated temperature‐dependent potential RuBP regeneration rate as described in Falge et al. (1997).

As indicated above, rather than using the notation of Vc_max_ at the leaf level, Vc_uptake_ is referred to as the estimate of the maximum rate of carboxylation capacity at the canopy level when fitting the model to EC data. Estimated Vc_uptake_ is extracted by minimizing the sum of residual least squares, that is, the Levenberg–Marquardt algorithms of the PV‐WAVE routine in the comparison of model predictions with EC observations at half hour intervals. The light utilization efficiency, alpha, can also be estimated from the EC data as an additional fitting parameter. In this study, however, we assumed alpha to be proportional to Vc_uptake_ following Owen et al. (2007). The relationship of alpha and Vc_uptake_ is

(8) $$\text{alpha}=\min\left(0.0008{\mathrm{Vc}}_{\text{uptake}},0.06\right)$$

Vc_uptake_ estimation was carried out on a daily basis over the course of the growing season for each crop data set. We determined that the predicted Vc_uptake_ was occasionally unrealistically high during the early growing season (>200 μmol m‐2 leaf area s‐1) when LAI was low (<1), but GPP was positive (>50 μmol m‐2 leaf area s‐1). This results from errors in the flux determination or in LAI estimation, values that are critical in the analysis of CO_2_ uptake (it may occur, for example, if the flux footprint is different from the area used in LAI estimation). In order to eliminate artificially high estimates of Vc_uptake_, the current work includes only flux data where LAI is >1 in the model fitting procedure. More information on the physiologically based model can be found in Owen et al. (2007).

#### 2.2.2 Physiological parameter estimates

The leaf physiological parameters applied as constants and those controlling temperature dependencies were obtained in previous studies on leaf physiology. These values are identified and shown in Table 1 (from Falge et al., 1996; Harley & Sharkey, 1991; Sala & Tenhunen, 1996; Tenhunen et al., 1990). These parameters describe temperature and light dependencies and response of stomata (Tenhunen et al., 1990). Gross primary production (GPP) is calculated with the single‐layered canopy sub‐model as described in section 2.2.1. Inputs to the model are half‐hourly global radiation, air temperature, relative humidity, and precipitation. Matrices for all meteorological drivers are prepared previous to analysis runs (estimated in separate routines and stored outside of the model) and are input to the model according to the half‐hourly simulation time step. Of primary concern in this study was estimation of a single key parameter, Vc_uptake_, that describes change in overall canopy CO_2_ uptake capacity.

Seasonal variation of Vc_uptake_ was estimated via model fitting with the detailed canopy sub‐model and EC GPP data (Owen et al., 2007). The method was performed using the functions NLINLSA and NONLINREGRESS of PV‐WAVE statistical program package. These functions used a modified Levenberg–Marquardt algorithm that is a method for minimizing a sum of weighted squared residuals to solve nonlinear parameter problems, such as estimation of Vc_uptake_ (PV‐WAVE IMSL Mathematics Reference, https://help.perforce.com/pv‐wave/2017.0/pvwave_online_help/; Transtrum & Sethna, 2012). A common least‐squares minimization is expressed as:

(9) $$C\left(\theta \right)=\frac{1}{2}\sum_{m=1}^{M}r_{m}{\left(\theta \right)}^{2}$$

where *N* ≤ *M*, *r*: RN‐>RM is an M‐dimensional nonlinear vector function of *N* parameters θ. Function *r*_*m*_(θ) followed the form

(10) $$r_{m}\left(\theta \right)=\frac{\left(y\left(t_{m},\theta \right)-y_{m}\right)}{\sigma_{m}}$$

θ is the parameter, *y* (*t*_*m*_, θ) is a model of the observed data, *y*_*m*_, that depends on unknown parameters θ, one or more independent variables t, and uncertainty in observed data, σ. The terms in Equation 10 are known as the residuals, the parameter values that minimize *C*(θ) are known as the best fit parameters. Consistent seasonal trends in the key physiological parameter describing CO_2_ uptake capacity, Vc_uptake_, are found for functional crop types, for example, root crops and rice as a grain crop (Li et al., 2010), which aids in parameterization according to the land use.

#### 2.2.3 Leaf area index estimates

Leaf area index (LAI) is the key parameter related to plant phenology and measured during the growing season at the rice paddy sites, Haean, Mase, and El Saler. Since measured LAI has in previous studies been shown to be correlated with spectral reflectance (Fan et al., 2008; Pontailler et al., 2003; Stenberg et al., 2004; Xiao et al., 2002), NDVI was applied to estimate LAI. The relationship between vegetation indices and LAI is generally applicable or “universal” rather than site specific, individual sites were examined by pooling the data for all sites. Lee et al. (2016) suggested a new method for estimation of the seasonal course in LAI that focuses on and requires only the identification of the time at which maximum NDVI is attained; we adapted the method based on Lee et al. (2016).

## 3 RESULTS

### 3.1 Physiological parameter estimation

The development of the LAI is an adaptation from Lee et al. (2016), using the seasonal course of LAI of rice paddies determined by NDVI and applying the consistent development curve method. The estimated LAI (Lee et al., 2016, figure 8) effectively captures the increasing and decreasing phases of and magnitudes of LAI.

**FIGURE 1:**
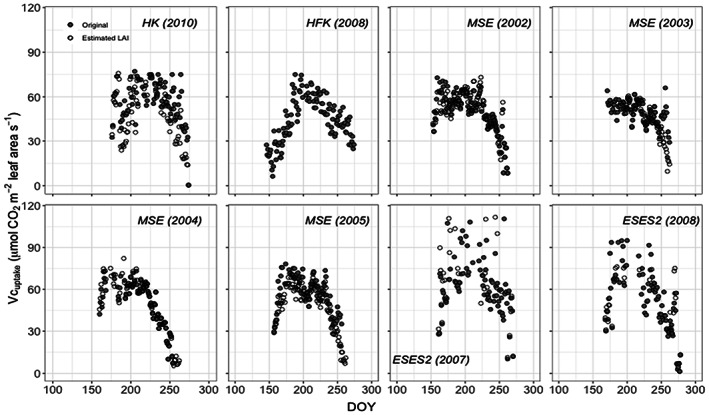
Seasonal change of Vc_uptake_ obtained with measured LAI (“Original,” black closed circle) and with the consistent development LAI‐NDVI model (“estimated LAI,” gray closed circle) of rice paddy sites for years indicated. HK = Haean (S. Korea), HFK = Haenam (S. Korea), MSE = Mase (Japan), and ESES2 = El Saler‐Sueca (Spain).

Having established a reliable method that allows estimation of the seasonal course of LAI across sites, the influence of differences between measured versus predicted LAI on estimation of carboxylation capacity was tested. The seasonal change of carboxylation capacity (Vc_uptake_) is illustrated in Figure 1 as determined by both the measured LAI (Vc_uptake_org_) and the estimated LAI (Vc_uptake_). The seasonal pattern of Vc_uptake_org_ increased rapidly and, in a characteristic manner after planting, reached a maximum in most cases at ca. DOY 170 to 180 and then decreased slowly during further development and later senescence of the rice sites. It should be noted that the results apply only during the period where LAI >1.0 and where Vc_uptake_ can be determined with relatively high certainty as discussed under methods.

Approximate maximum values based on measured LAI was 88 μmol CO_2_ m^−2^ leaf area s^−1^ at HK during 2010; 78 in 2002, 70 in 2003, 83 in 2004, and 83 in 2005 at MSE; and difficult to determine for ESES2 due to the high degree of scatter in the data. These results are influenced by the consistency of measurements or homogeneity at the measurement sites. The most homogeneous results were obtained from MSE, where it appeared that carboxylation capacity in 2004 and 2005 was slightly higher than during 2002 and 2003. The values for Vc_uptake_org_ also depend on the exact determination of LAI, for example, the methods that each research group uses during their measurements. Since these influences are impossible to remove, one must conclude that the maximum for Vc_uptake_org_ may be ca. 80 to 85 μmol CO_2_ m^−2^ leaf area s^−1^. The consistent results at MSE demonstrate that Vc_uptake_org_ after maximum may decrease differently depending on climate or other factors. Data from MSE in 2004 and 2005 exhibited a more rapid decrease than in 2002 and 2003 during the senescence period. The scattered data from ESES2 must occur due to day to day changes in the measured fluxes, since LAI changes from day to day were essentially zero. The cause of such changes is unclear.

**FIGURE 2:**
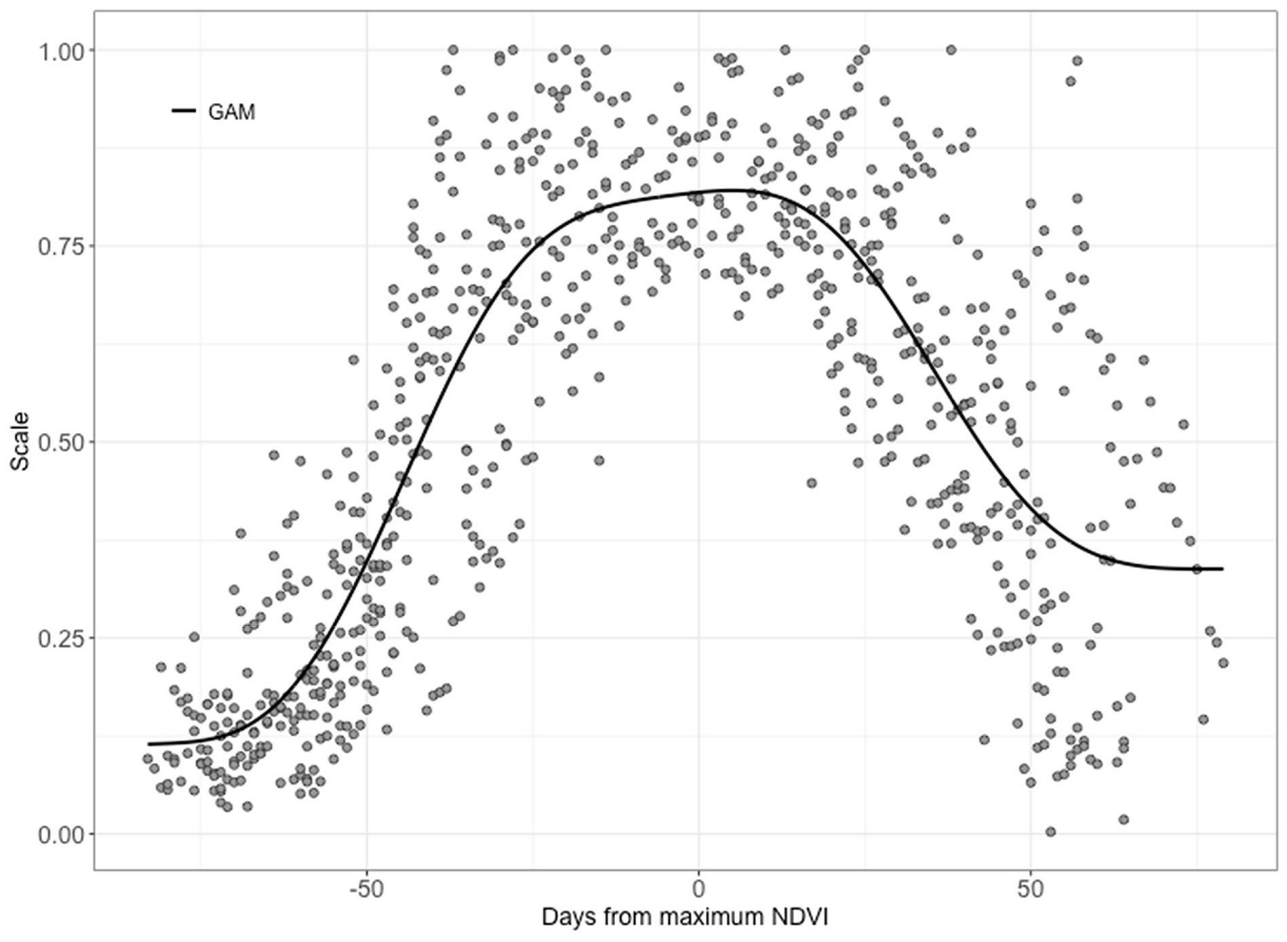
A scaled general seasonal curve for Vc_uptake_ utilizing data from all rice paddy sites. The scaling results by dividing estimated Vc_uptake_ by maximum Vc_uptake_ for each site and year. The black closed circle indicates the relationship between the scaled Vc_uptake_ and DOY based on time shifts according to maximum NDVI. The solid line indicates the general seasonal curve determined by generalized addictive model (GAM).

Comparisons of Vc_uptake_ obtained with LAI from the consistent phenology development model as well as the commonly used exponential‐curve based model are given in Table 2. Vc_uptake_ obtained with the consistent development model agreed better with the values obtained with measured LAI with respect to *R* ^2^, RMSE and the correspondence with the 1:1 relationship, except at the HK site. Results at HK were, however, also quite good with *R* ^2^ of 0.82, RMSE of 7.33, and CV was 16%. The high values of *R* ^2^ (above 0.82), low RMSE (below 9.93), and low CV (3%–25%) reflect the agreement illustrated in Figure 2 and further demonstrate that LAI obtained by the consistent development method is useful in the estimation of GPP at the study sites.

**TABLE 2:** Statistics for the linear correlation between Vc_uptake_org_ and Vc_uptake_. *a* is a slope, *b* (μmol CO_2_ m^−2^ s^−1^) is an intercept, *R* ^2^ is the coefficient of determination, RMSE (μmol CO_2_ m^−2^ s^−1^) is root mean square error, and CV is coefficient of variation.

		Vc_uptake_ by consistent phenological development					Vc_uptake_ by exponential model				
Site	Year	*a*	*b*	*R* ^2^	RMSE	CV (%)	*a*	*b*	*R* ^2^	RMSE	CV (%)
HK	2010	0.87	−0.92	.82	7.33	15.97	0.95	0.74	.85	1.75	3.94
MSE	2002	0.96	0.74	.95	1.24	2.93	1.08	0.85	.89	4.58	10.82
MSE	2003	0.95	−0.06	.86	2.14	5.63	0.9	2.83	.83	2.2	5.4
MSE	2004	1.01	0.07	.99	0.51	1.29	1.05	5.35	.75	6.96	22.45
MSE	2005	0.87	0.74	.94	5.83	13.18	1.14	0.37	.93	6.41	17.06
ESES2	2007	1.3	−4.98	.93	9.93	24.63	1.31	1.71	.74	17.37	39.53
ESES2	2008	1.09	0.03	.96	3.2	11.1	1.15	3.13	.84	8.59	27

### 3.2 GPP estimation with the best‐fit model

**FIGURE 3:**
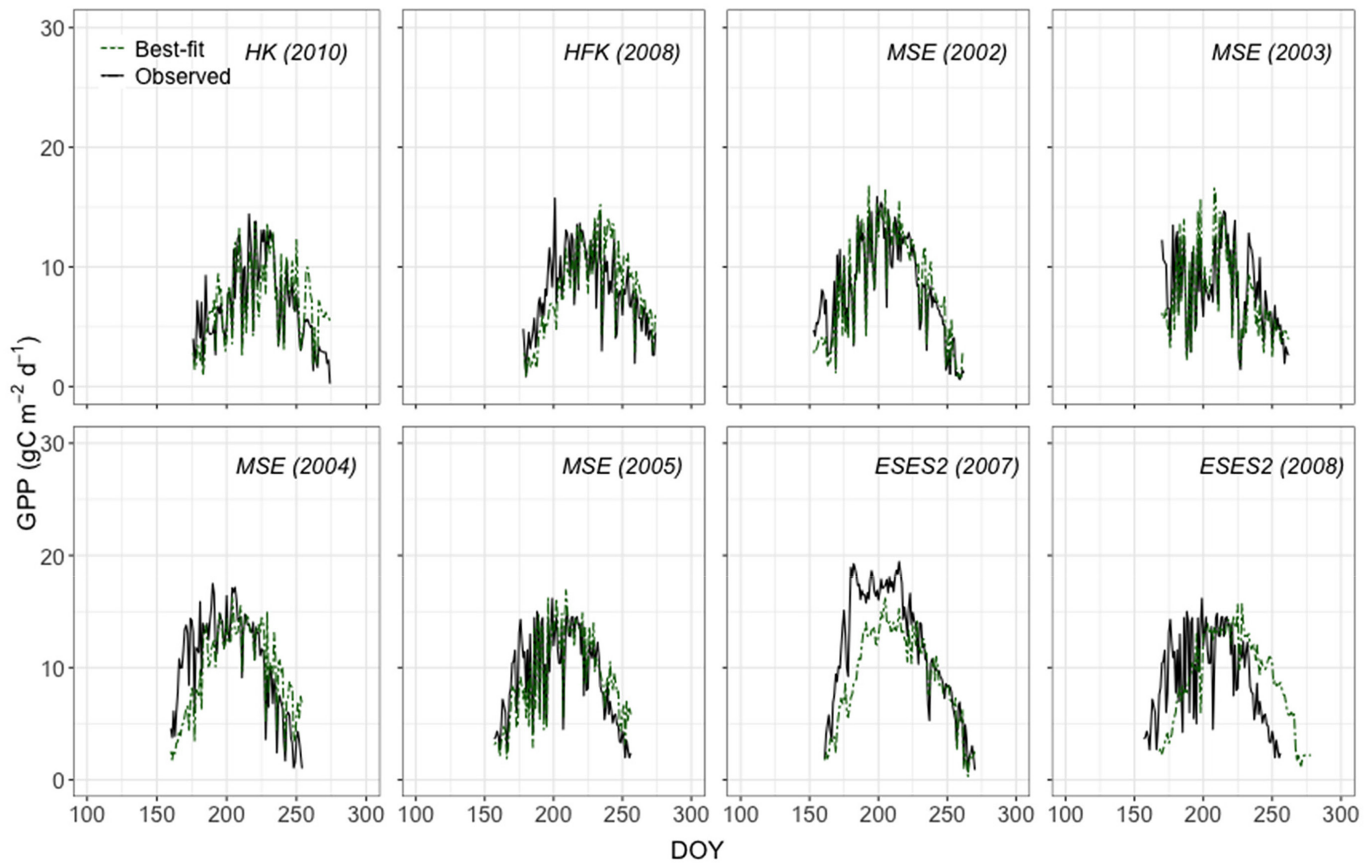
Daily GPP estimation with the general seasonal curve model for Vc_uptake_ (dark green dashed line) and observed GPP (black solid line) of rice paddy sites for the years indicated. HK = Haean (S. Korea), HFK = Haenam (S. Korea), MSE = Mase (Japan), and ESES2 = El Saler‐Sueca (Spain).

Daily GPP as reproduced by the model, which was LAI and Vc_uptake_ both dependent on consistent development curves, is in Figure 3. Accumulated observed GPP during the growing season varied from 672 gC m^−2^ d^−1^ to 1294 gC m^−2^ d^−1^, although the period for comparison varied slightly. Carbon uptake appeared to increase from Korea, to Japan, and to Spain due to different climate conditions. Modeled values ranged from 670 gC m^−2^ d^−1^ to 1020 gC m^−2^ d^−1^ with *R* ^2^ above 0.79, RMSE below 3.48, CV above 38.02% and modeling efficiency (MF) between 0.92 and 0.72 (Table 3). Simulated GPP was in general under‐estimated as expected due to the remaining difficulties in estimating Vc_uptake_ in dependence on NDVI. Deviations from observation over the course of the season at each site reflect the differences found for observed and predicted Vc_uptake_ as represented in Figure 3 or in Table 3. Although the alternative method based on consistent phenological development did not lead to an improvement in GPP prediction, further development of the response curve shown in Figure 2 may lead to better success as discussed later.

**TABLE 3:** Summary of observed GPP and modeled GPP obtained with the linear regression model for Vc_uptake_ from NDVI at all rice paddy sites with mean, standard deviation (STD), accumulated GPP (acc.GPP), difference between simulated GPP and observed GPP (Diff.), slope (a), intercept (b), determination coefficients (*R* ^2^), root mean square error (RMSE), coefficient of variation (cv), and modeling efficiency (MF).

Site	Year	Observed GPP			Modeled GPP			Period (DOY)	Diff. (%)
		Mean	STD	Acc.GPP	Mean	STD	Acc.GPP		
HK	2010	6.01	8.6	672	5.99	8.7	670	114	0
HFK	2008	7.11	9.15	780	7.84	10.41	860	96	10
MSE	2002	7.21	9.73	897	6.57	9.43	817	109	−9
MSE	2003	7.12	9.4	749	7.26	10.2	763	92	2
MSE	2004	9.19	11.82	987	7.94	11.18	852	94	−14
MSE	2005	8.03	10.5	908	7.31	10.08	826	99	−9
ESES2	2007	10.41	12.89	1294	7.81	11.32	971	109	−25
ESES2	2008	9.22	11.95	1157	8.12	11.4	1020	110	−12

Site	Year	a	b	*R* ^2^	RMSE	CV (%)	MF		
HK	2010	0.91	0.54	0.8	0.77	65.4	0.79		
HFK	2008	1.01	0.67	0.79	0.75	68.25	0.72		
MSE	2002	0.93	−0.15	0.93	0.94	38.02	0.92		
MSE	2003	1.03	−0.08	0.9	0.31	44.91	0.88		
MSE	2004	0.87	−0.09	0.85	2	51.21	0.84		
MSE	2005	0.91	−0.01	0.9	1.2	42.32	0.9		
ESES2	2007	0.82	−0.72	0.87	3.48	51.48	0.83		
ESES2	2008	0.88	−0.02	0.86	1.82	50.5	0.85		

## 4 DISCUSSION

In developing a methodology for estimating GPP at a large scale, it was determined that inputting remotely sensed information into vegetation canopy models would help to achieve more accurate estimates. However, the reflectance observed by remote sensing is influenced by both physiological parameters and vegetation phenological development (i.e., changing canopy structure). The relative importance of these in determining vegetation indices is largely unknown. While in this study separation of their effects was not attempted, an approach of consistent development curves is used to relate NDVI stepwise both to LAI change and to physiological change over the course of the season. Seasonal changes in LAI and in the key physiological model parameter Vc_uptake_, which reflects canopy carboxylation capacity at a given LAI (described as dependent on the vegetation index NDVI). The relative successes and failures in these efforts are discussed below.

### 4.1 Use of NDVI to estimate Vc_uptake_ for rice paddies

The eddy covariance methodology (EC) applied at rice paddy sites quantifies seasonal changes in GPP in relation to prevailing meteorological conditions (Kwon et al., 2010). By fitting the PIXGRO canopy model routine with flux data and with known LAI and meteorological conditions on a daily basis, an estimated seasonal time course for the key physiological parameter Vc_uptake_ (i.e., canopy carboxylation capacity) is obtained. Owen et al. (2007) obtained such estimates for many EC monitoring sites by assuming a constant LAI at the observed maximum level. They carried out their analysis with constant LAI because measured time courses for LAI (periodic measurements) did not exist at many of the study sites. While an assumption of a constant LAI at maximum level may allow a more complete use of data from EC study sites and in a way lead to reasonable spatial models for GPP, the objective of the current study is to use remote sensing first to determine structural change (the seasonal course of LAI). This requires comparing patterns for changes in canopy physiology for different crops (i.e., accurate seasonal patterns) for Vc_uptake_. At the beginning of this study, use of constant LAI was also examined, but seasonal LAI gave significantly better predictions for GPP (results not shown). In recent literature, various methods have been employed for GPP estimation, such as eddy covariance (EC) methods, process‐based models, and satellite remote sensing. While these methods have made significant advancements, there are still challenges in accurately estimating GPP in agroecosystems like rice paddies due to the incompatibility of spatial scales of flux measurements and satellite imagery resolution, as well as the influence of local climate, water availability, and varied management practices (Wattenbach et al., 2010; Yu et al., 2017). The results of this study are significant because they address these challenges by integrating multiple data sources and techniques, providing a more accurate and comprehensive approach for GPP estimation in rice paddies. This improved accuracy has potential applications for better understanding the impacts of climate variability on agricultural productivity, informing crop management decisions, and ensuring global food security. Additionally, the findings of this study may contribute to the development of more effective strategies for optimizing GPP and crop yield under varying environmental conditions.

A further consideration is that Vc_uptake_ patterns should be related to seasonal change in leaf carboxylation capacity as determined by other methods such as with leaf cuvette experimentation. The use of a seasonal course in LAI should help to bring together information from both EC and leaf level eco‐physiological studies.

### 4.2 Limitations of remotely sensed vegetation indices

A number of shortcomings remain in the use of satellite vegetation indices to estimate GPP. First, the uncertainties in NDVI may be caused by missing or bad data, or systemic errors in obtaining daily smoothed NDVI. Secondly, NDVI is not sensitive enough to capture rapid changes in crop development during an initial growth period (Wang et al., 2005). Similar results were found in this study for HK in 2010, MSE in 2003, and 2004. Thirdly, NDVI is relatively insensitive when LAI is over 4 (Brantley et al., 2011), leading to large predicted changes in LAI in derived regressions when NDVI changes during the middle of the season. Most of the rice sites exhibited an over‐ and/or underestimation of predicted LAI under NDVI regression. Since accurate LAI estimation is a key element in calculating GPP (Brantley et al., 2011), continued research focused on improving such estimates is essential in future studies, the consistent development approach must be explored further. Finally, LAI measurements were carried out every two or 3 weeks at eddy covariance sites. This infrequent sampling of LAI results in the tendency to miss important phenological events during the early growing season, which is critical to predicting the total GPP of the growth season.

Further studies are required in more detail and with accurately sampled spatial data in agricultural ecosystems with various crop types in order to calibrate LAI and physiological parameters such as Vc_uptake_ for use in models for GPP. With such studies, the restriction where eddy covariance data, GPP, and Vc_uptake_ can only be evaluated in scenarios where LAI >1.0 can be improved.

## 5 CONCLUSION

This study provides insights with respect to achieving improved methods for assessing GPP in rice paddy ecosystems through the use of process‐based models and remote sensing. The MODIS product provided by NASA underestimates and poorly represents GPP, especially in croplands (Gitelson et al., 2012; Yan et al., 2009; Zhang et al., 2008). By following a different methodology, MODIS vegetation indices are shown to be effective in estimating paddy rice phenology, LAI, and the simplified physiological parameter Vc_uptake_ at local site‐specific scale. In the case of paddy rice, a generalized model was developed to estimate GPP under various climate conditions and at geographically widely separated locations.

This study suggests methods for development of a “best‐fit model” to estimate GPP by bridging satellite information from VIs and ground observations. The best‐fit model constructed here, which used VIs to estimate phenology (LAI) and physiological parameter (Vc_uptake_) based on consistent development curves, provided accurate seasonal values for GPP with acceptable statistical values (Table 3), although limited so far to periods where LAI >1.0. Photosynthetic capacity in relation to VIs changes over the course of a growing season together with the carbon‐to‐nitrogen ratio of leaves in the plant canopy. Nitrogen in foliage determines vegetation's investments into chlorophyll and rubisco (Bonan, 2015), such as machinery for photosynthetic processes. This may decrease over the course of the season with little influence on reflectance properties. Where data were available, this study found that the carbon‐to‐nitrogen ratios, often accompanied changes in vegetation indices. These types of physiological changes (reflecting the relationship between Vc_uptake_ and NDVI) may account for the hysteresis effects that were observed in particular data sets.

Improvements in the modeling approach require that more detailed biological supplementary information should accompany EC studies in the future (e.g., frequent spatial samplings of crop canopy structures, local on‐the‐ground measurements of VIs, monitoring of physiological characteristics such as C/N ratios, chlorophyll content). On the one hand, detailed studies must be designed to support an extension of the approach described here, moving away from empiricism and linking remote sensing to the biological processes being observed. Ultimately, however, the estimation of crop GPP should be possible and should be undertaken without the need for additional field surveys, since these cannot be conducted on a large scale.

## FUNDING INFORMATION

Funding was provided by National Institute of Forest Science (FE100‐2022‐04‐2023).

## CONFLICT OF INTEREST STATEMENT

The authors declare no conflict of interest.

## Data Availability

Data derived from public domain resources.
